# Whole Blood Transcriptome Analysis Reveals the Correlation between Specific Immune Cells and Septicemic Melioidosis

**DOI:** 10.1155/2021/6166492

**Published:** 2021-10-13

**Authors:** Ke Xu, Dahua Xu, Hua Pei, Yunfan Quan, Jun Liu, Li Yin, Xuexia Li, ShenTian, Kongning Li, Qianfeng Xia

**Affiliations:** ^1^Key Laboratory of Tropical Translational Medicine of Ministry of Education and School of Tropical Medicine and Laboratory Medicine, Hainan Medical University, Haikou, Hainan, China; ^2^Key Laboratory of Tropical Translational Medicine of Ministry of Education and College of Biomedical Information and Engineering, Hainan Medical University, Haikou 571199, China; ^3^Department of Clinical Laboratory, The Second Affiliated Hospital, Hainan Medical University, Haikou, China; ^4^School of Basic Medicine and Life Sciences, Hainan Medical University, Haikou, Hainan, China

## Abstract

Melioidosis is a serious infectious disease caused by the environmental Gram-negative bacillus *Burkholderia pseudomallei*. It has been shown that the host immune system, mainly comprising various types of immune cells, fights against the disease. The present study was to specify correlation between septicemic melioidosis and the levels of multiple immune cells. First, the genes with differential expression patterns between patients with septicemic melioidosis (*B. pseudomallei*) and health donors (control/healthy) were identified. These genes being related to cytokine binding, cell adhesion molecule binding, and MHC relevant proteins may influence immune response. The Kyoto Encyclopedia of Genes and Genomes (KEGG) analysis revealed 23 enriched immune response pathways. We further leveraged the microarray data to investigate the relationship between immune response and septicemic melioidosis, using the CIBERSORT analysis. Comparison of the percentages of 22 immune cell types in *B. pseudomallei* vs. control/healthy revealed that those of CD4 memory resting cells, CD8+ T cells, B memory cells, and CD4 memory activated cells were low, whereas those of M0 macrophages, neutrophils, and gamma delta T cells were high. The multivariate logistic regression analysis further revealed that CD8+ T cells, M0 macrophages, neutrophils, and naive CD4+ cells were strongly associated with the onset of septicemic melioidosis, and M2 macrophages and neutrophils were associated with the survival in septicemic melioidosis. Taken together, these data point to a complex role of immune cells on the development and progression of melioidosis.

## 1. Introduction

Melioidosis, a serious tropical infectious disease, frequently outbreaks in Southeast Asia and northern Australia. It is caused by the Gram-negative bacillus *Burkholderia pseudomallei* (*B. pseudomallei*), which inhabits in soil and surface water [1]. In some endemic areas, melioidosis is a major cause of pneumonia in adults [2] and lethal septicemia, and case fatality rates range from 10 to 50% [3]. Naturally acquired disease in human species is the result of *B. pseudomallei*-induced infection due to the pathogenic bacteria entry through broken skin, inhalation, or ingestion [4]. Certain environmental conditions (tropical storms, rainy season, etc.) and certain occupations (rice farming [5]) are known to the risk factors of the infection [3]. In addition, several internal risk factors cannot be overnighted. For instance, diabetes mellitus, type 2 diabetes in particular, is a common risk factor predisposing individuals to melioidosis with more than 50% patients with melioidosis having type 2 diabetes [6]. Also, immunosuppression appears to be another important risk factor, as 60–90% of patients with melioidosis had the history of immunosuppressive treatments [3]. Like other infection diseases, there are of acute (caused by infections from recent bacterial exposure) and chronic types. Nearly 85% of melioidosis cases are of acute type, and patients with acute melioidosis develop sepsis rapidly, namely, septicemic melioidosis. This condition is a life-threatening one with a death rate of 40% [1].

The immune system is a host defense system consisting of a variety of immune cells, organs, proteins, and tissues that protects against disease. As the immune system meets a pathogen, it primarily invokes the two immune responses with the involvements of T cell and B cell in the adaptive immune response as well as neutrophils and macrophages in the innate immune response. In melioidosis, the immune responses play a big part as well. Epidemiologically, immunosuppression is believed to predispose individuals to *B. pseudomallei* infection [7, 8]. The weight of evidence is that in the most cases, *B. pseudomallei* infection merely leads to subclinical condition as most of immunocompetent individuals can remove the infection without any medical intervention [9]. In the C57BL/6 mice cell model, the microbicidal activity against *B. pseudomallei* was significantly lower in PECM (peritoneal exudate cells macrophage identified by nonspecific esterase) and NAPEC (nonadherent peritoneal exudate cells that were assumed to be full of lymphocytes) cultures when compared to peritoneal exudate cell (PEC) cultures, suggesting that macrophage-lymphocyte interactions promoted the killing of *B. pseudomallei* [10]. In agreement with this finding, patients with acute melioidosis come through with elevated levels of and CD8+ and CD4+ T cells, whereas those dead were with decreased levels of these cells [11]. Nevertheless, the immune response is also likely to be destructive as exemplified excessive recruitment of neutrophils causing tissue damage [12]. It is highly likely that multiple immune cells are engaged in melioidosis. However, due to technical limitations, the previous studies are unable to fully investigate the multiple immune cells, with only a small bunch of selected immune cells or mixed ones without proper identifications being explored. Therefore, it is required to investigate multiple immune cells in melioidosis with new technique.

The whole blood can recapitulate the real immune status of individuals. Previous studies have shown that the whole blood samples collected from melioidosis patients being subjected to microarray-based profiling had been proved to be an instrumental approach to investigate the onset and development of melioidosis [13]. More importantly, Conejero et al. reanalyzed the above whole blood transcriptional microarray data and provided useful insight into melioidosis [14]. Thus, in the current study, we reanalyzed the whole blood transcriptional microarray data in an effort to determine whether immune cells, the key players in the core immune network, play a big part in septicemic melioidosis. Accordingly, to better understand multiple immune cells contributing to the immune response to the disease, an established computational approach (CIBERSORT), which is capable of evaluating 22 immune cell types, was employed. This study was to more thoroughly investigate the specific roles of the immune cells in septicemic melioidosis.

## 2. Material and Methods

### 2.1. Patients' Characteristics and Dataset Collection

The human whole blood microarray and the clinical data were obtained from NCBI GEO datasets, and the accession number was GSE13015. In the current study were 8 healthy donors (control/healthy), 12 patients with type 2 diabetes (type 2 diabetes), 9 patients recovering from melioidosis (control/recovery), and 40 patients with septicemic melioidosis (*B. pseudomallei*). In addition, 37 patients with sepsis caused by other organisms (1 *Acinetobactor baumannii*, 1 *Aeromonas hydrophila*, 4 *C. albicans*, 3 *Corynebacterium* spp., 8 *E. coli*, 1 *Enterococcus faecium*, 2 *Enterococcus* spp., 1 *K. pneumoniae*, 1 *S. pneumoniae*, 1 *Salmonella serotype* B, 2 *Salmonella* spp., 4 *Staphylococcus aureus*, 6 *Staphylococcus coagulase* negative, and 2 *Streptococcus* non A, B.) were included. With the limma package in R, differentially expressed genes were identified with absolute cutoff log2 fold change value greater than or equal to 0.9. The clinical data were shown in a previous research [13].

### 2.2. Enrichment Analysis of the Gene Ontology (GO) Function and Kyoto Encyclopedia of Genes and Genomes (KEGG) Pathway

The package of http://org.Hs.eg.db in R was used to convert the gene name to Entirez IDs. ClusterProfiler package was then used to enrich the network with GO enrichment and KEGG enrichment pathways [15]. The results with *p* values less than 0.05 were considered statistically significant.

### 2.3. CIBERSORT Analysis

CIBERSORT, as an instrumental tool, can precisely measure the relative levels of distinct immune cell types. It can characterize each immune cell type with a bulk gene expression signature consisting of around 500 genes. Here, the original CIBERSORT gene signature file LM22, defining 22 immune cell types, was applied for analyzing the dataset from the septicemic melioidosis. CIBERSORT metrics with Pearson correlation coefficient, CIBERSORT *p* value, and root mean squared error (RMSE) were measured for each sample [16].

### 2.4. Statistical Analysis

Difference between immune cell types was determined by Mann-Whitney *U*-test. Correlations among immune cell types and clinical features were determined by Spearman's rank correlation. A value of *p* < 0.05 was considered significant. The relevant parameters with spearman absolute *r* value > 0.5 were selected. The multivariate logistic regression analyses [17] were performed to identify potential risk factors for septicemic melioidosis. In order to clear the impact of multicollinearity of the covariates in the multivariate regression model [18], the Akaike information criterion (AIC) was therefore used for the further selection of parameters. Only those noncorrelated parameters (with AIC < 10) were included in the multivariate logistic regression analysis. The receiver-operating characteristic (ROC) curve was plotted [19] with the true-positive fraction (sensitivity) versus the false-positive (1-specificity). A value of the area under the curve (AUC) equal to 0.9–1.0 is regarded as a perfect prediction, while a value of 0.5 is equivalent to a random. An AUC value between 0.7 and 0.9 indicated a good prediction, whereas an AUC value between 0.5 and 0.7 indicates a bad prediction.

## 3. Results

### 3.1. Transcriptomic Analysis of Human Whole Blood

**Figure 1:**
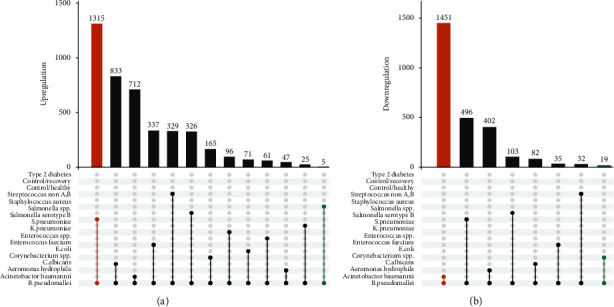
Upset plot depicting the numbers of the genes considered differentially expressed. (a) The upregulation of differentially expressed genes. There are multiple groups for comparison. (b) The downregulation of differentially expressed genes. The numbers of differentially expressed genes are indicated above. Type 2 diabetes vs. control/recovery, control/recovery vs. control/healthy, *B. pseudomallei* vs. *Acinetobactor baumannii*, *B. pseudomallei* vs. *Aeromonas hydrophila*, *B. pseudomallei* vs. *C. albicans*, *B. pseudomallei* vs. *Corynebacterium* spp., *B. pseudomallei* vs. *E. coli*, *B. pseudomallei* vs. *Enterococcus faecilum*, *B. pseudomallei* vs. *Enterococcus* spp., *B. pseudomallei* vs. *K. pneumoniae*, *B. pseudomallei* vs. *S. pneumoniae*, *B. pseudomallei* vs. *Salmonella serotype* B, *B. pseudomallei* vs. *Salmonella* spp., *B. pseudomallei* vs. *Staphylococcus aureus*, *B. pseudomallei* vs. *Staphylococcus coagulase* negative. Note: when the number of the differentially expressed genes was zero, it was remove from the plot. For instance, the results of *B. pseudomallei* vs. *Staphylococcus coagulase* negative, control/recovery vs. control/healthy, and type 2 diabetes vs. control/healthy were null.

As diabetes mellitus, type II diabetes in particular, is believed to be a general risk factor predisposing individuals to melioidosis, and this allows us to hypothesized that differentially expressed genes might be present among healthy donors (control/healthy), patients with type 2 diabetes (type 2 diabetes), and patients who had recovered from melioidosis (control/recovery). It is extremely important as the differentially expressed genes can constitute a promising gene signature with which to alert individuals who are susceptible to *B. pseudomallei* infection. Our findings, however, showed no significant differential expression between control/healthy and type 2 diabetes, as well as between control/healthy and control/recovery (Figure 1). This corroborates previous findings, in which control/healthy dataset cannot be separated from type 2 diabetes counterpart according to unsupervised clustering analysis [13], suggesting that background type II diabetes predisposing individuals to melioidosis may not map to significant transcriptional changes. Despite this, type 2 diabetes and control/recovery datasets were intended not to be integrated into the control/healthy counterpart for the subsequent analysis, as doing so could allow potential confounders to obscure the “real” effect. It has been documented that patients with septicemic melioidosis has a higher mortality rate as compared to the other pathogen-caused diseases [1]. We first determined whether there were distinct genes being differently expressed between the patients with septicemic melioidosis (designated as *B. pseudomallei*) and patients with other infections. As shown in Figure 1, a series of differential expression patterns were displayed. Of note, no significant differential expression between *B. pseudomallei* and *Staphylococcus coagulase* negative was found. Five genes were upregulated, and no gene downregulated in *B. pseudomallei* vs. *Staphylococcus aureus*. It appears that no overlap between genes that are differentially expressed between *B. pseudomallei* vs. other infections could be found. More importantly, no significant overlap between genes that are differentially expressed between *B. pseudomallei* and other infections was found, even when *Staphylococcus aureus* and *Staphylococcus coagulase* negative were removed. Analogue to our findings, Sangwichian et al. found that two other bacterial infections were falsely identified as *B. pseudomallei* by real-time PCR, suggesting that it is difficult to differentiate melioidosis from other pathogenic infections by transcriptional analysis [20].

### 3.2. Enrichment Analysis of the GO Function and KEGG Pathway

**Figure 2:**
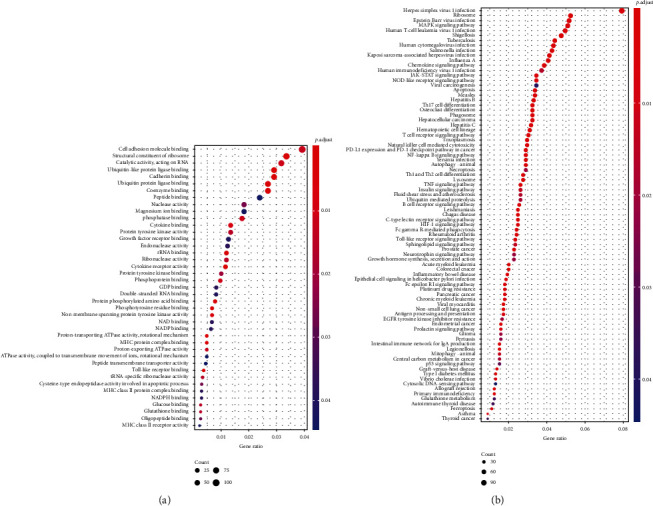
Analysis of gene enrichments. (a) Enriched Gene Ontology (GO) biological process identified by analyzing the differentially expressed genes in *B. pseudomallei* vs. control/healthy. Those with *p*.adjust.value < 0.05 were included. (b) Enriched KEGG pathways identified by analyzing the differentially expressed genes in B*. pseudomallei* vs. control/healthy.

A total of 3593 genes, of which 2149 were upregulated and 1444 downregulated, that were differentially expressed between *B. pseudomallei* and control/healthy were identified. Functional enrichment of Gene Ontology (GO) biological process annotations were identified among differentially expressed genes. They were mainly related to cytokine binding, cell adhesion molecule binding, Toll-like receptor binding, and MHC relevant proteins (Figure 2(a)). This suggests that multiple genes relevant to immune response were involved. We then reasoned that the biological pathways related to immune response may take a big part. Biological pathway analysis by Kyoto Encyclopedia of Genes and Genomes (KEGG) was performed. The results with an adjusted *p* value < 0.05 are shown in Figure 2(b). Apparently, the most genes were enriched on the pathways related to immunology, such as Human T-cell leukemia virus 1 infection, chemokine signaling pathway, human immunodeficiency virus 1 infection, JAK-STAT signaling pathway, Th17 cell differentiation, T cell receptor signaling pathway, natural killer cell-mediated cytotoxicity, PD-L1expression and PD-1 checkpoint pathway in cancer, NF-kappa B signaling pathway, Th1 and Th2 cell differentiation, TNF signaling pathway, B cell receptor signaling pathway, rheumatoid arthritis (type III hypersensitivity), Fc gamma R-mediated phagocytosis, Toll-like receptor signaling pathway, acute myeloid leukemia, inflammatory bowel disease, chronic myeloid leukemia, antigen processing and presentation, graft-versus-host disease, allograft rejection, primary immunodeficiency, and autoimmune thyroid disease. These findings suggest that the immune response may stand to influence *B. pseudomallei* infection.

### 3.3. Distribution of Immune Cells in the Septicemic Melioidosis

**Figure 3:**
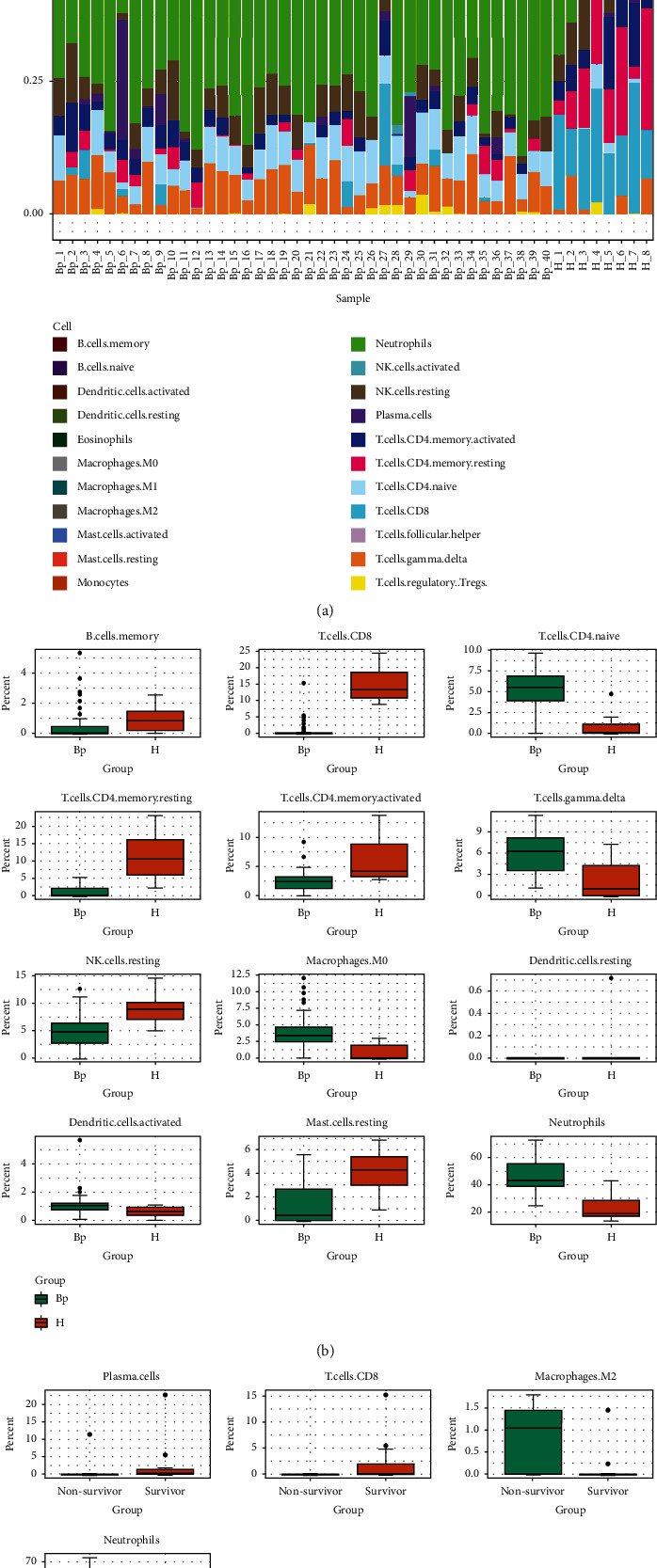
Correlation between the immune cells and the onset and development of septicemic melioidosis. (a) Stacked bar plot describing in immune cell compositions of patients with septicemic melioidosis and healthy control, derived using the CIBERSORT analysis, was carried out. Each bar represents percent fractions for the cell types, with colors representing the different cell types. (b) Boxplots depicting proportions of relevant immune cell types between patients with melioidosis and healthy control. Mann-Whitney *U*-test was performed, and *p* < 0.05 was allowed to be plotted. (c) Boxplots depicting proportions of relevant immune cell types between survivors and nonsurvivors with melioidosis. Mann-Whitney *U*-test was performed, and *p* < 0.05 was allowed to be plotted.

Since immune cells are essential players in the immune response, our focus was primarily on them [21], the CIBERSORT was employed for exploring the proportion of each immune cell type between *B. pseudomallei* and control/healthy. The distribution of cell fractions was illustrated in Figure 3(a). In order to determine whether certain cell types were associated with septicemic melioidosis, the Mann-Whitney *U*-test was used to measure significant difference in the proportions of the immune cells between *B. pseudomallei* and control/healthy. Among the 22 immune cell types, those with *p* less than 0.05 were plotted. As expected, the proportion of neutrophils was high in *B. pseudomallei*, when compared with control/healthy, coinciding with the previous result that excessive neutrophils play a damaging role in the patients with melioidosis [12]. In contrast to control/healthy, the proportion of CD8+ T cells was markedly low in *B. pseudomallei*, which is analogue to the findings published by Jenjaroen et al. that a lower CD8+ T cell level was observed in the worsen outcomes caused by *B. pseudomallei* infection [11]. The proportion of naive CD4+ T cells, a quiescent T cell subpopulation, was high in *B. pseudomallei* when compared with control/healthy, whereas that of CD4 memory resting cells and CD4 memory activated cells were low in *B. pseudomallei*. In addition, the proportion of B memory cells in *B. pseudomallei* was low too. These findings suggest that adaptive immune response is likely to be suppressed in patients with septicemic melioidosis. We also observed that the proportions of M0 macrophages, resting macrophage, and gamma delta T cells, an unconventional T cell subpopulations that are the key components of innate immune response, were high in *B. pseudomallei*, whereas that of NK resting cells was low, suggesting the interplay between *B. pseudomallei* infection and innate immune response was complicated (Figure 3(b)).

Since the immune cells were implicated in septicemic melioidosis, we then investigated whether the immune cell types were associated survival in septicemic melioidosis. As shown in Figure 3(c), only four immune cell types were selected. As expected, the proportion of neutrophils was high in nonsurvivors, when compared with survivors. The higher proportion of M2, an immune suppressor cell subpopulation, was present in nonsurvivors, whereas the proportions of plasma cells and CD8+ T cells were lower. These findings suggest that the adaptive immune response was overwhelmingly suppressed in severe septicemic melioidosis.

### 3.4. Correlation between the Immune Cells and the Onset of Septicemic Melioidosis

**Figure 4:**
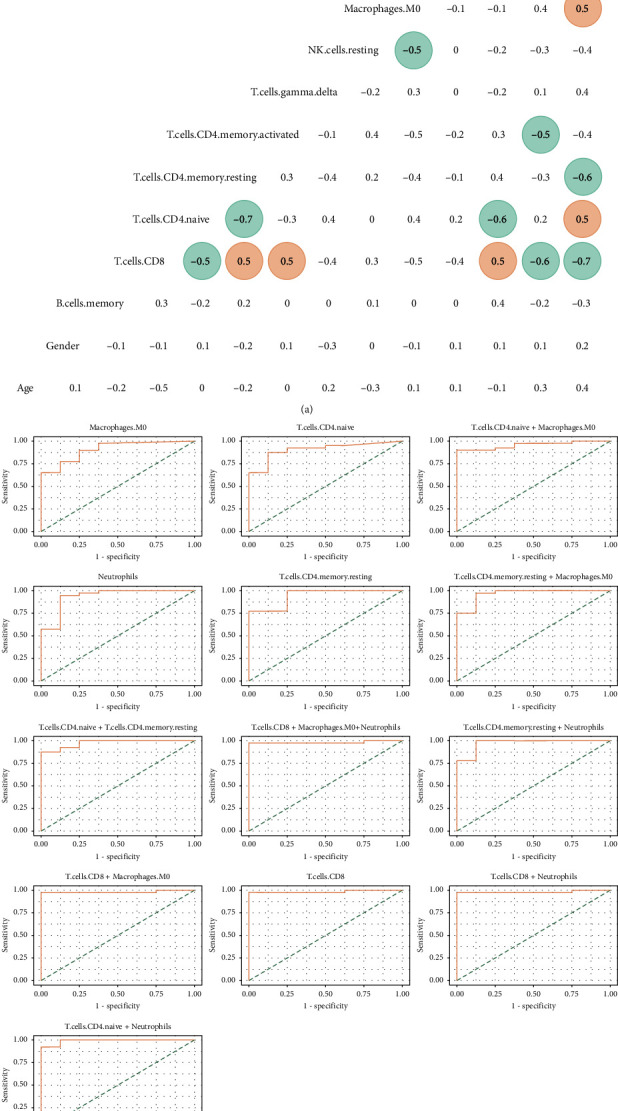
Further confirmation of the correlation between selected immune cell types and the onset of septicemic melioidosis. (a) Heatmap depicting correlation among selected immune cell types and several clinical features in the patients with melioidosis, with absolute *r* value > 0.5 highlighted with color round marks. (b) Receiver-operating characteristic curves depicting the combinations of selected immune cell types between patients with melioidosis and healthy control. The curves with at least one immune cell type's *p* value less than 0.05 were allowed to be plotted. AUC (area under the curve), specificity, and sensitivity were shown in the following brackets. M0 macrophages (0.91, 1.00, 0.65); naive CD4+ cells (0.91, 0.88, 0.88); naive CD4+ cells + M0 macrophages (0.96, 1.00, 0.9); neutrophils (0.94, 0.88, 0.95); resting CD4 memory T cells (0.94, 1.00, 0.78); resting CD4 memory T cells + M0 macrophages (0.97, 0.88, 0.98); naive CD4+ cells + resting CD4 memory T cells (0.98, 1.00, 0.88); CD8+ T cells + M0 macrophages + neutrophils (0.98, 1.00, 0.98); resting CD4 memory T cells + neutrophils (0.97, 0.88, 1.00); CD8+ T cells + M0 macrophages (0.98, 1.00, 0.98); CD8+ T cells (0.98, 1.00, 0.98); CD8+ T cells + neutrophils (0.98, 1.00, 0.97); naive CD4+ cells + neutrophils (0.99, 1.00, 0.93).

We next investigated the correlations between the selected immune cells and several clinical features, of which all serve as parameters for correlation analysis. Those selected parameters (with absolute *r* value > 0.5 highlighted with color round marks) were included in the multivariate logistic regression analysis. As shown in Figure 4(a), the neutrophils, M0, and naive CD4+ T cells were positively correlated with melioidosis, while the resting CD4+ T cells and CD8+ T cells were negatively correlated with melioidosis. The other immune cell types, along with age and gender were not correlated with melioidosis (Figure 4(a)). Therefore, the neutrophils, M0, naive CD4+ T cells, resting CD4+ T cells, and CD8+ T cells were selected for multivariate logistic regression. Multiple parameter models were then generated based on the full combinations of these selected parameters. The models with at least one parameter's *p* value < 0.05 were allowed to be proceeded with ROC curve analysis, and the results were illustrated in Figure 4(b). The ROC curve analysis gave AUCs from 0.91 to 0.99. It is safe to say that each model was promising, as each of AUC values is greater than 0.7. Top 5 AUC values were given 0.98 from CD8+ T cells + M0 macrophages, 0.98 from CD8+ T cells + M0 macrophages, 0.98 from CD8+ T cells, 0.98 from CD8+ T cells+ neutrophils, and 0.99 from naive CD4+ cells + neutrophils, respectively. Of note, the two-parameter models of naive CD4+ cells + neutrophils increased the AUC marginally from 0.91 (one parameter model of naive CD4+ cells) and 0.99. All the more than two parameter models but CD8+ T cells + M0 macrophages + neutrophils had been excluded presumably because of multicollinearity. These findings suggest that several models are able to predict the onset of septicemic melioidosis.

### 3.5. Correlation between the Immune Cells and Survival of Patients with Septicemic Melioidosis

**Figure 5:**
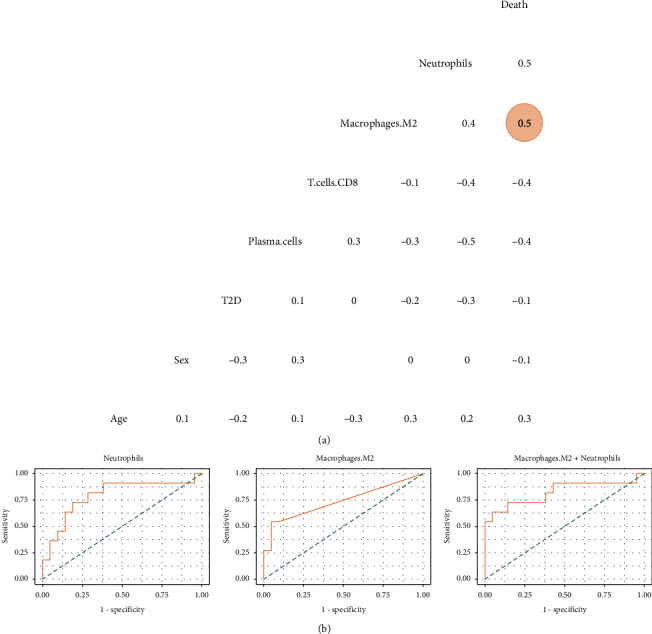
Further confirmation of the correlation between selected immune cell types and the development of septicemic melioidosis. (a) Heatmap depicting correlation among selected immune cell types and several clinical features in the patients with melioidosis, with absolute *r* value > 0.5 highlighted with color round marks. (b) Receiver-operating characteristic curves depicting the combinations of selected immune cell types between survivors and nonsurvivors with melioidosis. The curves with at least one immune cell type's *p* value less than 0.05 were allowed to be plotted. AUC (area under the curve), specificity, and sensitivity were shown in the following brackets. Neutrophils (0.79, 0.81, 0.73); M2 macrophages (0.74, 0.95, 0.55); M2 macrophages + neutrophils (0.82, 0.95, 0.64).

As several immune cells were associated with the development of melioidosis, we then asked whether most of these immune cells were implicated in the survival of patients with septicemic melioidosis. The correlation analysis was carried out as described above. Surprisingly, type II diabetes was not relevant to the patient survival, with very low absolute *r* value (*r* value = -0.1) (Figure 5(a)). Only M2 was eligible for the following the logistic regression analysis. Because the absolute correlation score of neutrophils was marginally less than 0.5, and neutrophils were reckoned as a relevant parameter. Thus, neutrophils was added to the logistic regression analysis, regardless of its correlation result. ROC curve analysis gave AUCs from 0.74–0.82. The two-parameter model of M2 macrophages + neutrophils largely improved the one parameter model of M2 macrophages (Figure 5(b)).

## 4. Discussion

This study was to establish correlation between septicemic melioidosis and the levels of multiple immune cells. First, the genes that were differentially expressed between patients with septicemic melioidosis (*B. pseudomallei*) and health donors (control/healthy) were identified. These genes were linked to cytokine binding, cell adhesion molecule binding, and relevant MHC functions. The Kyoto Encyclopedia of Genes and Genomes (KEGG) pathway findings revealed 23 enriched immune response pathways. We further leveraged the microarray data to investigate the relationship between immune response and melioidosis. CIBERSORT analysis was performed. Comparison of the levels of 22 immune cell types in *B. pseudomallei* vs. control/healthy revealed that the levels of CD8+ T cells, CD4 memory resting cells, CD4 memory activated cells, and B memory cells were low, whereas those of M0 macrophages, gamma delta T cells, and neutrophils were high. The multivariate logistic regression analysis further revealed that CD8+ T cells, M0 macrophages, neutrophils, and naive CD4+ cells were strongly associated with septicemic melioidosis, and M2 macrophages and neutrophils were associated with severe septicemic melioidosis. Together, these data point to a complex interplay of mechanisms underlying the effects of specific immune cells on the onset and development of septicemic melioidosis.

In melioidosis, *B. pseudomallei* can multiply within macrophages without activating a bactericidal response [22]. On the other hand, activated macrophages by inflammatory cytokines display improved killing of *B. pseudomallei* [23]. Although increasing evidence favors the important roles played by macrophages in melioidosis, the roles of specific macrophage subsets in the disease remain unclear. In general, macrophages are classified into nonactivated (M0), proinflammatory (M1), and anti-inflammatory (M2) subsets, each of which plays a distinctive role in the inflammation. We demonstrated that the level of M0 macrophages was high in patients with melioidosis, and the greater level of M2 macrophages was biased towards severe septicemic melioidosis. Our results suggest that conversion from M0 to M2 macrophages may simply reflect the onset and development of septicemic melioidosis (Figures 4 and 5). To the best of our knowledge, this result represents a novel and striking example in which shifts between macrophage subsets are correlated with the development and progression of melioidosis. In agreement with our finding, the previous studies of macrophage subsets on the other intracellular bacterial pathogens (IBPs) demonstrated that M2 macrophages were a comfortable replication niche for *Salmonella* and *Brucella* strains [24, 25]. M2 macrophages also favored replication of *Chlamydia pneumoniae* [26]. The results of the present study, as well as those of other researchers, support the view that M2 macrophages are favorable to IBP infections. Intriguingly, increasing level of M2 macrophages was associated with worse survival in lung cancer [27]. Therefore, further exploration of M2 macrophages may help improve the treatment for melioidosis.

We observed that patients with septicemic melioidosis exhibited the extremely low level of CD8+ T cells, when compared with healthy donors. And CD8+ T cells alone were able to predict the onset of septicemic melioidosis (Figure 4(b)). Moreover, an extreme level of CD8+ T cells was present in nonsurvivors with septicemic melioidosis. Our findings suggest that a low level of CD8+ are associated not only with the onset of septicemic melioidosis but also with death caused by septicemic melioidosis. Analogue to these, a decreased level of CD8+ T cells was found to be correlated with greater mortality [11]. This finding experimentally confirms the role played by CD8+ T cells in the disease as well. Jenjaroen et al. also found that the level of CD4+ T cells was elevated in survivors of melioidosis vs. nonsurvivors. This observation was not found in this study. Perhaps, the conflicting observations can be explained by difference in methodological approaches or the subsets of the patients. It should be noted that several T cell subsets were implicated in the onset of melioidosis, as measured by CIBERSORT, an algorithm that allows us to profile 22 immune cell types. In *B. pseudomallei* vs. control/healthy, the levels of CD4 memory resting cells, CD4 memory activated cells, CD8+ T cells, and B memory cells were elevated, whereas the level of naive CD4+ T cells was decreased (Figure 3(b)). More importantly, the levels of plasma cells and CD8+ T cells were lower in nonsurvivors with septicemic melioidosis than those in survivors **(**Figure 3(c)). These findings suggest that the adaptive immune response is likely to be suppressed in patients with septicemic melioidosis, and deteriorating adaptive immune response might lead to poor survival.

Immune checkpoint molecules are inhibitory receptors expressed on immune cells that result in immunosuppressive signaling pathways. These molecules are key for modulating the duration and magnitude of immune responses [28, 29]. Signaling through these molecules can deplete immune cells, especially T cells. T cell exhaustion is reflected by expression of immune checkpoint molecules, such as the expressions of programmed cell death protein 1(PD1) and cytotoxic T lymphocyte antigen 4 (CTLA4). Blockade of PD1 and CTLA4 favored T cell-mediated immune response. It has been well documented that several pathogens (such as genus *Plasmodium vivax*) and cancers lead to inhibitions on the immune cells via immune checkpoint proteins [30–32]. The data of Figure 2(b) showed that PDL1/PD-1 checkpoint pathway is involved in septicemic melioidosis, suggesting that, at minimum, significant change in gene expression levels of either PDL1 or PD-1 could be found. However, no change in the two genes' levels was significant. Of note, in addition to PDL1 and PD-1, there are other major immune checkpoint elements, such as LAG3, TIM3, TIGIT, CD96, BTLA, TNF5F14, GITR, and VISTA [33]. Among these major immune checkpoint elements, we found that LAG3, CD96, and BTLA were strongly overexpressed in patients with septicemic melioidosis (data not shown), suggesting that the depletion of the T cell subsets in septicemic melioidosis might be the result of overexpression of LAG3, CD96, and BTLA. Blockade of these elements might be promising and novel strategy for the treatment of septicemic melioidosis. Moreover, LAG3 was commonly coexpressed with PD1 [34]. Dual blockade of BTLA and PD1 improves antitumor immunity [35]. Blockade of CD96 and PD1-PDL1 was able to improve tumor control [36]. These lines of evidence suggest PD1 and PDL1 should still be taken into account in the future studies. The exclusion of the two genes in this study might be due to the cut-off fold change setting.

Apart from immune response, multiple differentially expressed genes had functions relating to glucose metabolism such as NAD binding, NADP binding, glucose binding, glutathione binding, and GDP binding (Figure 2(a)). In addition, the glutathione metabolism was identified in the KEGG analysis (Figure 2(b)). Although the role of glucose metabolism in melioidosis remains unclear, it has been found in other intracellular pathogenic bacteria. When growth factors are available, the growth factor-stimulated, proliferating immune cells take up glucose by several glucose transporters (GLUTs) to produce larger quantities of intermediary metabolites and NADPH. These molecules are brought into glutathione production and launch the aerobic glycosis, which is a metabolic hallmark for many fast proliferating cancers. Moreover, HIF-1 enhances transcription of GLUTs and most enzymes of the glycolytic pathway. P53 activated glutaminase 2 (GLS2) and this enzyme catalyzes glutamine. These above-mentioned might explain why many cancer-related pathways are implicated in septicemic melioidosis [37].

## 5. Conclusion

In conclusion, the findings of present study indicate the relationship between the specific immune cell types and onset and development of septicemic melioidosis. These findings broaden our view on the complex interplay between immune cells and *B. pseudomallei* infection and offer novel insights into the roles of immune cell types in septicemic melioidosis.

## Data Availability

The datasets in this study can be obtained in NCBI datasets. The accession number(s) can be found in this paper.
